# Editorial: Comprehensive insights into ANCA vasculitis: from immunopathology to treatment strategies

**DOI:** 10.3389/fmed.2026.2022704

**Published:** 2026-10-26

**Authors:** Ligia Gabrie, Alan D. Salama, Reza Zonozi

**Affiliations:** 1 Department of Rheumatology, Marqués de Valdecilla University Hospital, Santander, Spain; 2 Department of Renal Medicine, UCL Centre for Kidney and Bladder Health, Royal Free Hospital, London, United Kingdom; 3 International Kidney Vasculitis and Glomerular Center, Fairfax, VA, United States; 4 Nephrology Associates of Northern Virginia, Fairfax, VA, United States

**Keywords:** ANCA-associated vasculitis (AAV), autoimmunity, biomarkers, granulomatosis with polyangiitis (GPA), immunopathology, microscopic polyangiitis (MPA), precision medicine

Anti-neutrophil cytoplasmic antibody (ANCA)-associated vasculitides (AAV), comprising granulomatosis with polyangiitis (GPA), microscopic polyangiitis (MPA), and eosinophilic granulomatosis with polyangiitis (EGPA), are rare, potentially life-threatening autoimmune diseases characterised by necrotising small-vessel inflammation and marked clinical and biological heterogeneity (1). Although therapeutic advances have improved outcomes, management remains challenging because disease activity, irreversible damage, relapse risk, and treatment-related toxicity may coexist and evolve differently within individual patients (1, 2).

This Frontiers Research Topic brings together 11 original research articles, reviews, and case reports addressing complementary aspects of AAV biology. Collectively, they illustrate an increasingly individualised approach based on biological context, clinical phenotype, risk profile, and dynamic response to treatment.

One fundamental question remains regarding how autoimmunity is initiated and maintained. Like Goodpasture's original description of a pulmonary renal syndrome (which in retrospect may have represented a vasculitic process) following the influenza pandemic (3), the study by Gabrie et al. identified a transient increase in AAV incidence during the COVID-19 pandemic, from 22.4 cases per million in 2019 to 37.9 per million in 2021, with incidence returning towards pre-pandemic levels in 2022. Approximately 40% of patients diagnosed with AAV had experienced SARS-CoV-2 infection or vaccination within the three months preceding diagnosis. Although these findings are consistent with transient immune priming, the observational design does not establish causality and larger multicentre studies are needed. At a more mechanistic level, Chen et al. showed that MPO-specific B cells can be detected in healthy individuals, patients with active MPO-AAV, and patients in long-term remission, whereas their immunogenomic characteristics and antibody output differed between groups. Restricted B-cell receptor repertoires, greater clonotype overlap, and distinct MPO epitope recognition in patients with disease suggest that the qualitative characteristics of the autoimmune response may be more informative than the mere presence of autoreactive B cells or circulating ANCA.

This concept of qualitative immune dysregulation is further developed by García-Serrano et al., whose review describes alterations across multiple immune checkpoint pathways, including PD-1/PD-L1, CTLA-4, ICOS, CD40-CD40L, LAG-3, TIM-3, BTLA, and CD27. Several soluble and cellular checkpoint markers have been associated with disease activity, renal involvement, or treatment response, while lower soluble LAG-3 and higher soluble CD27 have been reported to predict rituximab treatment failure. These observations provide a potential bridge between immunopathology and clinically useful biomarkers, although their incorporation into routine decision-making remains to be established.

The clinical phenotype may also challenge conventional disease classification. Xie et al. highlight the diagnostic and therapeutic complexity of patients with systemic lupus erythematosus and renal biopsy-proven AAV, in whom overlapping manifestations can obscure the distinction between lupus nephritis and pauci-immune glomerulonephritis. Similarly, Wu et al. describe MPO-ANCA-associated hypertrophic pachymeningitis preceding renal involvement and subsequently evolving into a phenotype fulfilling criteria for MPA. The case also illustrates the potential role of individualised low-dose rituximab in refractory disease. These reports emphasise that AAV may manifest through organ-specific phenotypes that are not readily captured by conventional systemic disease categories.

The Research Topic also addresses a central practical problem: how to balance disease control against treatment-related harm. Wang et al. developed a nomogram incorporating age, albumin, cumulative prednisone exposure, and baseline systemic immune-inflammation and systemic inflammation response indices (SII and SIRI) to estimate the risk of infection during the first six months of immunosuppressive treatment. In their cohort, 41.6% of patients developed an infection, predominantly respiratory, and the model incorporating the five independent predictors showed better discrimination than the clinical model alone. Complementing this approach, Rathmann et al. found hypogammaglobulinaemia in 45% of rituximab-treated patients, with lower baseline IgG independently predicting its subsequent development. Hypogammaglobulinaemia was not associated with an increased risk of severe infection, supporting close immunoglobulin monitoring while illustrating the need to interpret laboratory abnormalities in their clinical context and to further investigate differences between cohorts.

The question of monitoring extends beyond conventional inflammatory markers and ANCA titres. Colina reviews an expanding range of circulating, urinary, and tissue biomarkers alongside advanced imaging techniques, including FDG-PET/CT, vessel-wall MRI, vascular ultrasound, and magnetic resonance angiography. These approaches may be relevant when clinical assessment, serology, and tissue activity provide discordant signals. However, an important distinction remains between biomarkers and imaging modalities with sufficient evidence for clinical use and promising proof-of-concept approaches requiring further validation. This distinction is essential if increasingly sophisticated tools are to improve rather than complicate clinical decision-making.

Finally, several contributions address how biological and clinical information might translate into treatment selection. Treppo et al. propose a phase-oriented framework distinguishing induction, consolidation, and maintenance, with therapeutic intensity informed by disease severity, organ involvement, histopathology, ANCA specificity, and patient-level vulnerability to toxicity. The small retrospective cohort reported by Ye et al. provides preliminary clinical experience with obinutuzumab in AAV, including its use in induction and maintenance, although its role remains uncertain. Emerging strategies including complement inhibition, FcRn blockade, and cellular therapies are also of considerable interest, but their evidence base remains heterogeneous.

The experience with rituximab itself illustrates why treatment response should not be considered binary. In a *post hoc* analysis of RITAZAREM, Prieto-González et al. identified a small subgroup with incomplete remission at month 4 (BVAS/WG = 1). All patients had previous PR3-ANCA positivity and ENT involvement, and relapse occurred more frequently during follow-up. However, the small sample size, *post hoc* design, and overlap with the broader PR3-positive GPA phenotype make these findings hypothesis-generating rather than evidence of a distinct rituximab-resistant phenotype (Prieto-González et al.). Together with the observations on B-cell biology and immune checkpoints, this raises an important question for future studies: whether treatment failure can ultimately be anticipated from biological features before clinical relapse becomes apparent.

Taken together, the 11 contributions reinforce the view that AAV cannot be fully understood or managed as a homogeneous disorder. The field is moving towards an approach in which biological characterisation, clinical phenotyping, risk stratification, and treatment selection are increasingly interconnected and individualised (Treppo et al.). However, these approaches are not all at the same stage of clinical development, and several proposed biomarkers and therapeutic strategies remain exploratory and require prospective validation. The challenge for the next phase of AAV research is therefore not simply to identify more biomarkers or therapies, but to determine which biological signals meaningfully change clinical decisions and improve outcomes (Colina). Such integration may ultimately allow treatment intensity to be better matched to disease biology, organ-specific risk, relapse potential, and treatment-related vulnerability, bringing personalised medicine in AAV closer to clinical reality.
